# Identifying delirium in Parkinson disease: A pilot study

**DOI:** 10.1002/gps.5270

**Published:** 2020-02-07

**Authors:** Rachael A. Lawson, Sarah J. Richardson, Alison J. Yarnall, David J. Burn, Louise M. Allan

**Affiliations:** ^1^ Institute of Neuroscience Newcastle University Newcastle upon Tyne UK; ^2^ Newcastle Institute for Ageing Newcastle University Newcastle upon Tyne UK; ^3^ Newcastle upon Tyne Hospitals NHS Foundation Trust Newcastle upon Tyne UK; ^4^ Faculty of Medical Science Newcastle University Newcastle upon Tyne UK; ^5^ Institute of Health Research University of Exeter Exeter UK

**Keywords:** delirium, incidence, inpatient, Parkinson disease, prevalence

## Abstract

**Introduction:**

People with Parkinson disease (PD) may be at increased risk of delirium and associated adverse outcomes. Delirium is an acute neuropsychiatric syndrome defined by confusion and inattention and is common in older adults. Previous studies may have underestimated the prevalence of delirium in PD because of overlapping symptoms, lack of awareness, and poorly defined criteria. We aimed to identify the prevalence and incidence of delirium in inpatients with PD.

**Measurements:**

Participants were inpatients with PD admitted over a 4‐month period. Delirium prevalence was classified using a standardised assessment at a single visit on the basis of the Diagnostic and Statistical Manual of Mental Disorders fifth edition (DSM‐5) criteria. To capture remaining time in hospital, incident delirium was diagnosed using detailed clinical vignettes and a validated consensus method.

**Results:**

Forty‐four PD patients consented to take part in the study, accounting for 53 admissions. Delirium prevalence was 34.0% (n = 18); reviewing participants over the duration of their hospital stay identified 30 (56.6%) incident delirium cases. The admitting team screened 24.5% for delirium, and delirium was documented in eight (14.8%) patients' medical notes. Patients with delirium were significantly older, had higher frailty scores, and had a longer hospital stay (*P* < .05 for all).

**Conclusions:**

Delirium is common in PD inpatients at admission and incidence increases during hospital stay, but delirium is commonly missed. Our results highlight the importance of screening for delirium throughout patients' stay in hospital. Future studies should consider frequent evaluation over the duration of hospital stay to identify emergent delirium during the admission.

1

**Key points**

- Prevalent delirium occurred in over a third of inpatient Parkinson admissions.
- Incident delirium increased to more than half of Parkinson admission.
- Delirium reporting was low both in the medical notes and in discharge letters.
- Delirium patients were older, frailer, and had a longer hospital stay.

## 2 INTRODUCTION

Delirium is a serious acute neuropsychiatric syndrome that is common in older adults admitted to hospital and is characterised by altered levels of consciousness, confusion, and impaired attention.^1^ In older adults, delirium has been associated with poorer outcomes, such as dementia,^2^ mortality, and institutionalisation.^3^ A recent systematic review suggested that people with Parkinson disease (PD) may be at increased risk of delirium.^4^ PD is a movement disorder^5^ predominantly affecting older adults and is associated with frequent nonmotor features.

The reported prevalence of delirium is 10% to 31% in medical inpatients,^3^ but in PD, delirium prevalence varies from 11% to 60% across inpatient studies.^4^ This variability is in part due to different operationalised criteria used to identify delirium, although in some studies, delirium was poorly defined or not at all. Additionally, delirium in PD may be associated with increased hospital stay compared with those without delirium,^6^ worsening motor symptoms, cognitive decline, and mortality.^7^ There are currently no studies reporting prevention or management of delirium in PD.^4^

Well‐designed studies with clearly operationalised delirium criteria are needed to accurately define delirium in PD and to better understand who may be at risk of developing delirium. This pilot study aimed to determine the prevalence and incidence of delirium in people with PD admitted to hospital and delirium assessment and reporting practices in clinical settings to inform future studies. Secondary aims include exploring differences between participants who did and did not have delirium.

## 3 METHODS

### 3.1 Participants

Between 26 March and 25 July 2018, all inpatients with PD admitted at Newcastle upon Tyne Hospitals were invited to take part in the study. Inclusion criteria comprised a diagnosis of PD according to UK Brain Bank Criteria^5^ made by a movement disorder specialist and a hospital admission during the recruitment period. Exclusion criteria comprised a diagnosis of nonidiopathic PD; the patient was near death; the patient lacked capacity to give informed consent and no appropriate consultee was available; or the patient had insufficient English to complete the assessments.

Written informed consent was sought from those with capacity; for patients who lacked, a personal consultee was identified who completed a consultee declaration form. This study was approved by the Yorkshire & the Humber‐Bradford and Leeds Research Ethics Committee.

### 3.2 Measures

Participants were assessed in a single research assessment while in hospital. Demographic and clinical information were collected. PD motor severity was assessed using the Movement Disorders Society Unified Parkinson Disease Rating Scale (MDS‐UPDRS) Part III and Hoehn and Yahr stage. Frailty was measured using the Clinical Frailty Scale (CFS).^8^

Prevalence of delirium was assessed prospectively using a standardised procedure using Diagnostic and Statistical Manual of Mental Disorders fifth edition (DSM‐5) criteria^1^ on the basis of the DECIDE study protocol.^2^ Prevalence was defined as the number of cases of delirium identified at the single research visit after participants were admitted to hospital during the 4‐month recruitment period. A collateral history was taken from participants' relative or carer to determine whether symptoms were an acute change or due to PD or cognitive impairment associated with PD. Delirium severity was assessed using the Memorial Delirium Assessment Scale (MDAS).^9^ Arousal was measured using the Observational Scale of Level of Arousal (OSLA),^10^ and agitation and sedation were measured using the modified Richmond Agitation and Sedation Scale (m‐RASS)^11^ and the Glasgow Coma Scale (GCS).^12^ Medical notes and patient discharge letters were reviewed for information as part of participants' standard care.

After discharge, participants' hospital notes were reviewed over their whole admission to determine incident cases of delirium to capture cases that may have resolved before the single research visit or developed afterwards. Incidence was defined as the total number of cases of delirium identified during participants' admissions to hospital during the 4‐month recruitment period. Incident delirium was diagnosed using a validated consensus diagnosis method described by Kuhn et al.^13^ Detailed clinical vignettes were compiled, and delirium symptoms were abstracted systematically. Authors (RAL, LMA, and AJY) independently rated each vignette as unlikely, possible, or probable delirium; disagreements were resolved by consensus.

### 3.3 Statistical analysis

Statistical analyses were performed using SPSS software (Version 24.0; SPSS, Armonk, NY: IBM Corp). Comparisons of means between two groups were performed using independent *t* tests or Mann‐Whitney *U* tests, depending on distribution. Pearson *χ* ^2^ tests were used to compare between‐group distributions of proportion. A Wilson 95% confidence interval (CI) was calculated for all proportions.

## 4 RESULTS

**Figure 1:**
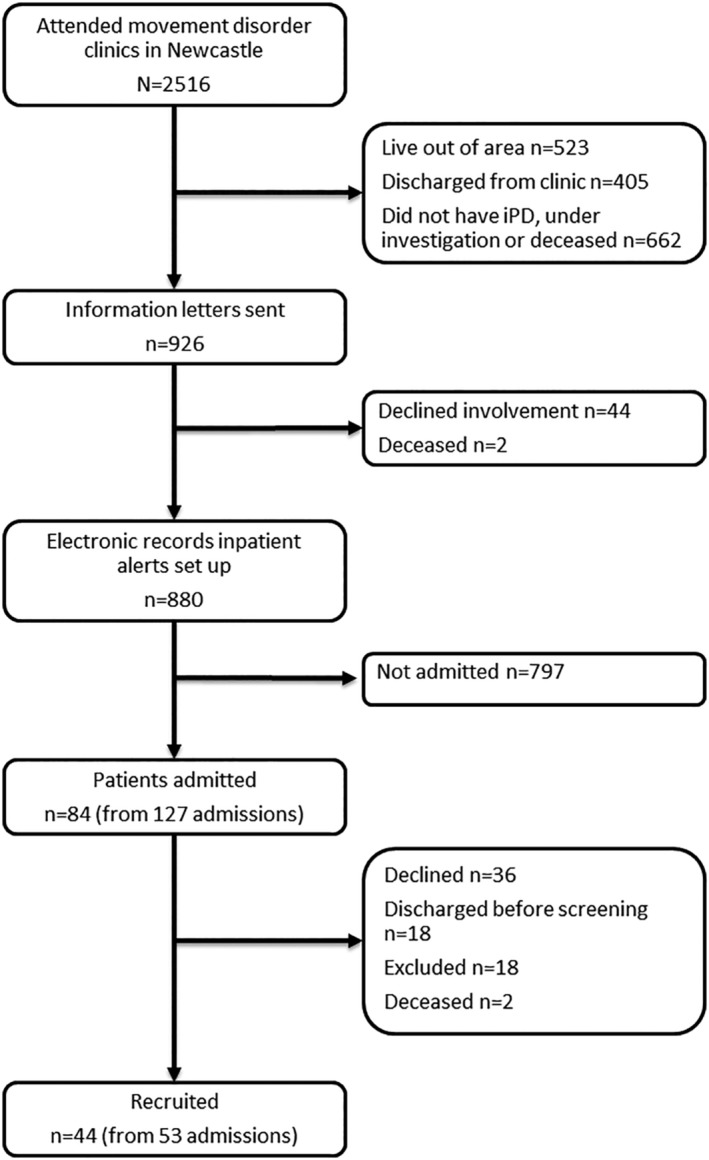
Flow diagram of recruitment. iPD, idiopathic Parkinson's disease

Over 4 months, 127 admissions from n = 84 people with PD were screened a mean of 28.1 ± 20.0 hours after admission (Figure 1). Forty‐four patients consented to take part in the study, accounting for 53 (47.1%) admissions.

Ages of PD patients admitted to hospital ranged from 46 to 99 (mean = 72.7 ± 12.6) years. Patients who declined were significantly younger than study participants (67.6 ± 15.8 vs 76.4 ± 9.7 years, respectively, *P* < .05). Study participants had a mean of 12.2 ± 3.2 years of education and PD duration of 6.2 ± 4.4 years (Table S1).

Of the 53 admissions, 90.6% (95% CI, 79.8%‐95.9%; n = 48) were emergency admissions (Table S2). Mean duration of hospital stay was 11.0 ± 12.7 days. Thirteen (24.5%; 95% CI, 0.149%‐37.6%) participants were screened for delirium by the admitting team, and three cases were identified as probable (n = 2) or possible (n = 1) delirium.

### 4.1 Delirium prevalence

The prevalence of delirium was 34.0% (95% CI, 22.7%‐47.4%; n = 18) at the single research assessment (Table 1). Mean time to assessment from admission was 39.7 ± 30.7 hours. Prevalent delirium PD patients had significantly higher Hoehn and Yahr stage, frailty score, and delirium severity measured by the MDAS and arousal score (OSLA) but lower GCS and agitation and sedation scores (m‐RASS, *P* < .05 for all, Table 1) compared with those without delirium at the single research assessment. No other significant differences were found (*P* > .05 for all).

**Table 1:** Comparison of characteristics of prevalent cases and incident cases of delirium in inpatients with PD

	Prevalent Delirium						Incident Delirium					
	No Delirium, n = 35		Prevalent Delirium, n = 18				No Delirium, n = 23		Incident Delirium, n = 30			
	Mean	SD	Mean	SD	*t*/*Z*	*P* Value	Mean	SD	Mean	SD	*t*/*Z*	*P* Value
Age	74.4	11	79.2	6.1	*t* = −2.1	**.044**	72.6	11.7	78.6	7.3	*t* = −2.2	**.037**
Education, y	12.3	3.3	12.1	2.7	*Z* = −0.1	.929	12.6	3.6	12	2.6	*Z* = −0.7	.497
MDS‐UPDRS III	49.7	14.7	56.4	17.7	*t* = −1.4	.158	48	15.1	55.3	15.3	*t* = −1.6	.118
Hoehn and Yahr stage	3.5	1	4.3	0.8	*Z* = −2.5	**.012**	3.4	1	4	1	*Z* = −2.2	**.025**
PD duration, y	6.3	4.5	6.8	4.9	*Z* = −0.5	.612	6.4	5.1	6.5	4.3	*Z* = −0.4	.693
No. of comorbidities	5.1	2.2	6.1	1.6	*Z* = −1.6	.104	5.1	2.5	5.6	1.7	*Z* = −0.7	.484
No. of medications	10.2	3.7	10.9	3.2	*Z* = −0.7	.46	9.8	3.8	10.9	3.2	*Z* = −1.4	.161
LEDD, mg/day	690.3	530.1	590.4	432.5	*Z* = −0.5	.598	617.9	492.5	685.9	507.1	*Z* = −0.4	.726
Hospital stay duration							6.2	6	14.7	15.1	*Z* = −2.9	**.004**
Clinical Frailty Scale	5.1	1.4	6.8	0.5	*Z* = −4.5	**<.001**	4.9	1.4	6.3	1.1	*Z* = −3.7	**<.001**
GCS total	14.5	1.3	12.1	1.9	*Z* = −4.9	**<.001**	14.5	1.2	13	2.1	*Z* = −3.2	**.002**
OSLA total	2.5	2.4	6.1	3.3	*Z* = −3.8	**<.001**	2.3	2.6	4.8	3.3	*Z* = −3.0	**.003**
m‐RASS	0.2	0.9	−1.1	2	*Z* = −2.2	**.03**	0.3	0.8	−0.6	1.8	*Z* = −1.2	.235
MDAS total	8.7	3.6	17.7	5.8	*t* = −6.0	**<.001**	8	3.6	14.7	6.2	*t* = −5.0	**<.001**

	n	%	n	%	*χ* ^2^	*P* Value	n	%	n	%	*χ* ^2^	*P* Value
Sex: male	23	65.7	12	66.7	0	.945	16	69.6	19	63.3	0.2	.635
Impaired on MoCA	9	25.7	1	5.6	6.4^a^	**.011**	6	26.1	4	13.3	0.4	.527
PD‐MCI	5	14.3	6	33.3	0.9	.763	4	17.3	7	23.3	0.8	.366
PDD	2	5.7	4	22.2	0.7^a^	.414	1	4.3	5	16.7	2.7	.102
Emergency admission							19	82.6	29	96.7	3.0^a^	.154
Delirium screened at admission							5	21.7	8	26.7	0.2	.679
Falls as inpatient							1	4.3	4	13.3	1.2^a^	.374
Increased package of care post admission							2	8.7	6	20	1.4^a^	.234

*Note*: Significant results are highlighted in bold.

Abbreviations: GCS, Glasgow Coma Scale; LEDD, Levodopa equivalent daily dose; MDAS, Memorial Delirium Assessment Scale; MDS‐UPDRS III, Movement Disorders Society Unified Parkinson's Disease Rating Scale Part III; MoCA, Montreal Cognitive Assessment; m‐RASS, modified Richmond Agitation and Sedation Scale; OSLA, Observational Scale of Level of Arousal; PD, Parkinson disease; PDD, Parkinson disease dementia; PD‐MCI, Mild Cognitive Impairment in Parkinson disease; SD, standard deviation.

a Fisher exact test.

### 4.2 Incident delirium

Reviewing participants' medical notes over the duration of their hospital stay^13^ identified 30 (56.6%, 95% CI, 42.3%‐69.1%) incident delirium cases comprising 10 cases of possible and 20 probable delirium. Only eight cases of delirium were documented (15.1%; 95% CI, 7.2%‐28.1%) in participants' medical notes, while only five cases of delirium were documented in discharge letters.

Patients with incident delirium (Table 1) were significantly older and frailer (*P* < .05 for both) compared with those who did not develop delirium during their hospital admission. Hospital duration was significantly longer in patients with incident delirium (mean of 14.7 ± 15.1 vs 6.2 ± 6.0 days, respectively, *P* < .01). Patients with incident delirium also had significantly higher delirium severity measured by the MDAS and arousal score (OSLA) but lower GCS and agitation and sedation scores (m‐RASS, *P* < .05 for all, Table 1) compared with those who did not develop delirium. No other significant differences were found in terms of demographic or clinical characteristic (*P* > .05 for all).

## 5 DISCUSSION

To our knowledge, this is the first prospective study to investigate prevalent and incident delirium in hospitalised patients with PD using standardised operationalised criteria. We found that prevalent delirium occurred in over a third of admissions, with incident delirium rising to more than half. However, delirium reporting was low both in the medical notes and in discharge letters.

Our results showed that delirium was common in inpatients with PD and higher than previously reported in older adults (23%)^14^ and medical inpatients.^3^ Previous studies have suggested that PD may be a risk factor for developing delirium. However, the reported prevalence of delirium in PD varies widely across studies, between 11% and 60% in inpatients.^4^ This may be due to the range of operationalised criteria previously used to identify delirium across studies. However, few studies explicitly addressed how each criterion was assessed. Although we found that delirium was common in PD, delirium reporting in medical notes and discharge letters was low. This is consistent with previous research.^15^ Since the completion of this study, however, hospital delirium screening guidelines have changed. Therefore, we would anticipate delirium reporting to be higher if this study was repeated.

Parkinson patients with delirium were significantly older, frailer, and had a longer stay in hospital compared with those without delirium. Increased age has been shown to be a risk factor for delirium in previous studies in PD.^6^ However, the link between frailty and delirium is not understood. There may be a dynamic relationship between the two, where frailty may be a risk factor for delirium, but delirium may be associated with cognitive decline and impeded physical recovery.^16^ Longer duration in hospital for patients with delirium in this study is consistent with studies both in older adults^3^ and in PD.^6^ This has an implication for increased health care costs.

Strengths of this pilot study include the prospective identification of point prevalent delirium and incident delirium using standardised procedures.^2^, ^13^ The inclusion of a collateral history was used to distinguish between participants' baseline and acute changes associated with delirium. Limitations include the small sample size at a single site, which limits the generalisability of results. The assessment used a single research visit, where delirium present at admission may have resolved before the research assessment or developed afterwards. However, we used a validated consensus diagnosis method^13^ to account for episodes of delirium missed from this single review. We did not include a non‐PD control group, which limits the interpretation of results. As the participants with delirium were frailer, it is not possible to tell whether frailty contributed towards the development of delirium independent of PD. However, there were no significant differences between those with and without delirium in terms of number of comorbidities or medications prescribed. Excluding those with delirium on admission using clinical vignettes, participants who later developed delirium had significantly higher frailty scores compared with those who did not develop delirium (data not shown). Future studies should consider using an age‐matched control group. Finally, the operationalised delirium criteria and assessments to aid delirium have not been validated in PD. Further work is needed to understand the sensitivity and accuracy of these assessments in PD.

## 6 CONCLUSION

In summary, delirium is common in inpatients with PD, although both delirium screening and documentation in medical notes were suboptimal. Our findings highlight the importance of delirium screening in inpatients with PD, both at admission and throughout hospital stay, but larger studies are needed with frequent evaluation to identify emergent delirium. Future studies should evaluate existing assessments and operationalised criteria for delirium for use in PD. A better understanding of delirium in PD and its presentation and accurate diagnostic criteria would have utility in future clinical trials to prevent or manage delirium.

## CONFLICT OF INTEREST

None declared.

## AUTHOR CONTRIBUTIONS

R.A. Lawson was involved with study design and conceptualisation of the study, coordination of the study, participant recruitment, data collection, statistical analysis, interpretation of data, and drafting of the manuscript. S.J. Richardson was involved with study design and conceptualisation of the study and review of the manuscript. A.J. Yarnall was involved with data collection, interpretation of data, and review of the manuscript. D.J. Burn was involved with study design and conceptualisation of the study, interpretation of data, and review of the manuscript. L.M. Allan was involved with study design and conceptualisation of the study, data collection, interpretation of data, and review of the manuscript.

## Supporting information

Table S1 Participant demographic and clinical characteristics

Table S2 Reasons for admission to hospital over four months

## Data Availability

Unidentifiable data may be shared on request.
