# Supplementary material for: Identifying delirium in Parkinson disease: A pilot study
Source: Int J Geriatr Psychiatry. 2020 Feb 7;35(5):547–52. doi: 10.1002/gps.5270 (PMC7186820; doi:10.1002/gps.5270)
Supplement: Supplementary file 1 — Table S1 Participant demographic and clinical characteristics [file GPS-35-547-s001.docx]

Supplementary Table 1: Participant demographic and clinical characteristics

| **Participant characteristics (n=44)** | | **Mean** | **SD** |
| --- | --- | --- | --- |
| *Age* |  | 76.4 | 9.7 |
| *Number of years of education* | | 12.2 | 3.2 |
| *PD duration (years)* | | 6.2 | 4.4 |
| *MDS-UPDRS III* | | 50.1 | 15.5 |
| *Hoehn and Yahr stage* | | 3.7 | 1.1 |
| *LEDD mg/day* | | 677.3 | 490.6 |
| *No. medications* | | 10.5 | 3.4 |
| *No. comorbidities* | | 5.3 | 2.1 |
| *Duration of cognitive impairment (years)* | | 1.5 | 1.1 |
|  |  | **n** | **%** |
| *Sex: male* | | 30 | 68.2 |
| *English first language* | | 42 | 95.5 |
| *Marital status* | |  |  |
|  | *Single* | 5 | 11.4 |
|  | *Married* | 21 | 47.7 |
|  | *Living with Partner* | 2 | 4.5 |
|  | *Separated* | 2 | 4.5 |
|  | *Divorced* | 3 | 6.8 |
|  | *Widowed* | 11 | 25.0 |
| *Place of residence* | |  |  |
|  | *Own house/flat* | 21 | 47.7 |
|  | *Rented flat* | 11 | 25.0 |
|  | *Relative's home* | 1 | 2.3 |
|  | *Sheltered accommodation* | 5 | 11.4 |
|  | *Residential care* | 3 | 6.8 |
|  | *Nursing home* | 3 | 6.8 |
| *Type of cognitive impairment* | |  |  |
|  | *No cognitive impairment* | 24 | 54.5 |
|  | *Impaired on MoCA* | 8 | 18.2 |
|  | *PD-MCI* | 7 | 20.5 |
|  | *PDD* | 5 | 11.4 |
| *Assistance with ADLs* | | 34 | 77.3 |
| *Carers* | | 31 | 70.5 |
|  | *Paid carer* | 16 | 36.4 |
|  | *Informal carer* | 17 | 38.6 |

PD = Parkinson’s disease; MDS-UPDRS III = Movement Disorders Society Unified Parkinson’s Disease Rating Scale Part III; LEDD = Levodopa equivalent daily dose; No. = Number, MoCA = Montreal Cognitive Assessment; PD-MCI = Mild Cognitive Impairment in Parkinson’s disease; PDD = Parkinson’s disease dementia.
