# Supplementary material for: Identifying delirium in Parkinson disease: A pilot study
Source: Int J Geriatr Psychiatry. 2020 Feb 7;35(5):547–52. doi: 10.1002/gps.5270 (PMC7186820; doi:10.1002/gps.5270)
Supplement: Supplementary file 2 — Table S2 Reasons for admission to hospital over four months [file GPS-35-547-s002.docx]

Supplementary Table 2: Reasons for admission to hospital over four months

| **Reason for admission (n=53)** | | **n** | **%** |
| --- | --- | --- | --- |
| *Emergency* | | 48 | 90.6 |
|  | *Fall* | 16 | 30.2 |
|  | *Pneumonia* | 4 | 7.6 |
|  | *Chest pain* | 3 | 5.7 |
|  | *Metabolic abnormality/dehydration* | 3 | 5.7 |
|  | *Heart attack* | 2 | 3.8 |
|  | *Pain* | 2 | 3.8 |
|  | *Accidental overdose* | 2 | 3.8 |
|  | *Delirium* | 2 | 3.8 |
|  | *Shortness of breath* | 2 | 3.8 |
|  | *UTI* | 2 | 3.8 |
|  | *Diarrhoea/vomiting* | 2 | 3.8 |
|  | *Medication reaction* | 1 | 1.9 |
|  | *Constipation* | 1 | 1.9 |
|  | *Syncopal episode* | 1 | 1.9 |
|  | *Catheter removal/Replaced* | 1 | 1.9 |
|  | *Urinary retention* | 1 | 1.9 |
|  | *Upper GI bleed* | 1 | 1.9 |
|  | *Pancreatitis* | 1 | 1.9 |
|  | *PEG complication* | 1 | 1.9 |
| *Elective* | | 5 | 9.4 |
|  | *DBS* | 2 | 3.8 |
|  | *Duodopa trial* | 1 | 1.9 |
|  | *Arthroplasty* | 1 | 1.9 |
|  | *Lumbar decompression* | 1 | 1.9 |

UTI = Urinary tract infection; GI = Gastrointestinal; PEG = Percutaneous endoscopic gastrostomy; DBS = Deep brain stimulation.
